# Real‐world titration, persistence & weight loss of semaglutide and tirzepatide in an academic obesity clinic

**DOI:** 10.1111/dom.70004

**Published:** 2025-08-05

**Authors:** Jason M. Samuels, Fei Ye, Rebecca Irlmeier, Heidi Silver, Gitanjali Srivastava, Matthew Spann

**Affiliations:** ^1^ Department of Surgery Vanderbilt University Medical Center Nashville Tennessee USA; ^2^ Department of Biostatistics Vanderbilt University Medical Center Nashville Tennessee USA; ^3^ Department of Public Health Sciences, Sylvester Comprehensive Cancer Center University of Miami Miami Florida USA; ^4^ Division of Gastroenterology, Hepatology, and Nutrition, Department of Medicine Vanderbilt University Medical Center Nashville Tennessee USA; ^5^ Department of Veterans Affairs Tennessee Valley Healthcare System Nashville Tennessee USA; ^6^ Division of Diabetes, Endocrinology & Metabolism, Department of Medicine Vanderbilt University School of Medicine Nashville Tennessee USA; ^7^ Vanderbilt Weight Loss Vanderbilt University Medical Center Nashville Tennessee USA

**Keywords:** antiobesity drug, GLP‐1 analogue, incretin therapy, real‐world evidence

## Abstract

**Aims:**

Trials of the Glucagon‐like Peptide‐1 Receptor Agonists (GLP1RAs) found mean weight losses of 15%–21%, yet realworld dose titration and persistence remain suboptimal, limiting effectiveness. This study aims to determine real‐world titration, persistence and effectiveness of GLP1RAs in patients managed within a multidisciplinary obesity clinic.

**Materials and Methods:**

This is a singlecentre, retrospective cohort study of patients seen in a multidisciplinary obesity clinic at an academic medical centre from January 2022 to December 2024. Consecutive patients aged 18–75 years enrolled in a ‘no cost to patient’ Medical Weight Loss Bundle program who received ≥1 prescription fill of semaglutide or tirzepatide. Of 2855 enrollees, 2306 (81%) received at least one GLP1RA prescription. The primary outcome was persistence with GLP1RA therapy (continuous prescription fills without a gap ≥84 days). Secondary measures included titration adherence and percentage change in body weight.

**Results:**

Among 2306 patients (median age 46.0 years, Interquartile Ratio [IQR] 38.0–55.0; 85% female; 68% White, 28% Black, 4% Hispanic), with median persistence 10.7 months (IQR 5.4–16.3). Semaglutide was used by 1614 (70%), tirzepatide by 117 (5%), and both agents by 575 (25%). Of semaglutide users, 81% escalated to ≥1 mg and 23% to 2.4 mg; of tirzepatide users, 75% received ≥10 mg and 28% received 15 mg. Among patients persistent for ≥6 months, median weight loss was 9.4% (IQR 6.0%–13.4%); for those persistent for ≥12 months, median weight loss was 14.4% (IQR 9.5%–20.5%).

**Conclusions:**

GLP1RA persistence and dosetitration adherence were moderate but weight loss approximated that seen in clinical trials, supporting real‐world effectiveness.

## 1 BACKGROUND

The STEP and SURMOUNT trials demonstrated that semaglutide and tirzepatide produce marked efficacy in obesity management, achieving mean total body weight reductions of 15% and 21%, respectively.^1^, ^2^ Given that over 40% of the U.S. population is affected by obesity, these findings have driven the rapid adoption of these therapies, which now constitute a substantial proportion of national prescriptions.^3^, ^4^ However, the extent to which these outcomes translate to real‐world settings remains unclear. Successful intervention necessitates gradual titration to maximally tolerated doses—a process often challenging to implement at scale within overburdened primary care systems.^5^ These agents are also associated with high rates of gastrointestinal adverse events,^6^, ^7^ which can compromise tolerability and precipitate premature discontinuation.^8^, ^9^, ^10^ Consequently, real‐world outcomes may not achieve the efficacy observed in clinical trials—a discrepancy well documented with other therapeutics.

Emerging data from European cohorts indicate that the real‐world effectiveness of Glucagon‐like peptide‐1 receptor agonists (GLP1RAs) may fall short of trial‐reported outcomes,^9^, ^11^, ^12^ potentially due to lower rates of dose escalation and abbreviated treatment durations.^10^ These patterns may reflect provider inexperience with established titration protocols, patient intolerance at higher doses, or supply chain constraints that limit access to maintenance regimens.^10^ However, existing investigations are constrained by heterogeneous patient populations—including individuals lacking obesity‐specific indications (e.g., Type 2 Diabetes) —and by insufficient clinical record granularity to disentangle prescribing practices from outcomes.

In this study, we leverage integrated electronic health records and pharmacy claims data from a multidisciplinary academic weight loss centre to evaluate real‐world prescribing patterns, adherence, tolerability and effectiveness of GLP1RAs (specifically semaglutide and tirzepatide) in a ‘no cost to patient’ environment. By examining dose‐titration trajectories, treatment persistence, and weight change patterns in a large cohort exclusively prescribed semaglutide or tirzepatide for obesity, we provide a detailed assessment of real‐world utilization patterns and their correlation with clinical effectiveness, distinguished by the inclusion of obesity‐specific indications, robust clinical records data, and integration of both Electronic Health Records (EHR) and claims data. Our findings aim to investigate the differences between real‐world and clinical trial outcomes, specifically timeliness and frequency of successful drug, discontinuation rates and impacts on weight.

## 2 MATERIALS AND METHODS

### 2.1 Data source

Data were obtained from the EHR and insurance claims repository at a single academic medical centre. Patients were identified by participation in the Medical Weight Loss Bundle program, and medical record numbers were used to link prescription fill data to corresponding EHR records. Data collection spanned from January 3, 2022, to February 25, 2025. The index date was defined as the date of the first GLP1RA prescription fill (semaglutide or tirzepatide), as indicated by the creation of a bill in the bundle clearing house. This study conforms with the recommendations of the STROBE guidelines for observational studies.^13^, ^14^

### 2.2 Data quality and linkage validation

Data underwent standard quality checks for completeness and internal consistency. Records with missing or unmatched EHR‐claims linkage were excluded. Prescription‐filled data were validated against medication prescribing entries and pharmacy claims to confirm actual dispensing. Fill data were assumed to reflect active medication use unless evidence of medication discontinuation was documented in provider notes or billing claims.

### 2.3 Study design, population and clinical setting

This is a single‐centre, retrospective, observational study conducted at a multidisciplinary academic medical weight loss clinic. Patients were eligible for inclusion if they: (1) were aged 18–75 years, (2) were enrolled in the Medical Weight Loss Bundle insurance add‐on, and (3) had received at least one prescription for semaglutide or tirzepatide for the indication of obesity treatment.

### 2.4 Description of medical weight loss bundle programme

The medical weight‐loss focused value‐based care bundle (MWLB) was developed to capture the total cost of care related to a comprehensive medical weight loss programme over a 12‐month period. Services included office visits with medical weight loss providers (physicians and advanced practice providers trained in Obesity Medicine), dietitians specialized in medical weight loss, weight‐loss focused mental health counsellors, access to exercise physiologists, dietary education materials, relevant laboratory and diagnostic testing, necessary specialty referrals, and all prescribed pharmaceuticals. Participants in the MWLB received these services without co‐pay or out‐of‐pocket expense. MWLB was made available for employers as a benefit for employees and their families. Of note, participating employers had gym benefits available for all employees. Employees and their families were not eligible for participation in the MWLB programme if they were actively participating in another value‐based care bundle (such as maternity or joint replacement bundles). Participants in this study were enrolled from January 3, 2022, through December 6, 2024.

### 2.5 Dietary and physical activity guidance

Participants in the MWLB were recommended to follow a diet higher in protein (25%–30% of daily of calories), moderate in carbohydrates (40% daily calories), and lower in fat (30%–35% daily calories) split across three daily meals. Meal plans and daily caloric needs were tailored by dietitians to balance macronutrients and individual needs. Participants were offered a referral for a medical fitness evaluation within our institution and exercise prescription as per standardized recommendations for 150 minutes per week, which is encouraged.

### 2.6 Prescribing definitions

Persistence was defined as continuous prescription fills without a gap exceeding three prescribing periods (defined as 28 days each). Adherence to the recommended titration schedule was assessed based on package insert guidance for semaglutide^15^ and tirzepatide,^5^ which recommend dose escalation every 4 weeks, with the goal of reaching the maximum recommended dose by the sixth prescription. Crossover between semaglutide and tirzepatide was considered continued persistence with GLP1RA therapy. Patients who unenrolled from the bundle during the study period were censored as non‐persistent due to loss of insurance coverage for GLP1RA and loss of subsequent prescription fill data. We stratified GLP1RA dosing into ‘low‐dose’ and ‘high‐dose’, using semaglutide <2 mg and tirzepatide <10 mg to define ‘low‐dose’.

### 2.7 Patient characteristics

Baseline demographic and clinical characteristics, including age, sex, race, ethnicity and pre‐existing comorbidities, were extracted from the EHR at the time of bundle enrollment. Comorbid conditions marked as resolved prior to enrollment were not considered active during the study period. Baseline weight was extracted using the closest in‐clinic recorded weight within 30 days of the index date. Implausible values (e.g., weight < 30 kg) were excluded. Percent total weight loss (%TWL) was calculated as Baseline Weight−WeightatTimepoint/Baseline Weight×100.

### 2.8 Study outcomes

The primary outcome was persistence with GLP1RA therapy. Secondary outcomes included: (1) adherence to recommended titration protocols, (2) maximum dose achieved, (3) transition between GLP1RA agents, (4) transitions from GLP1RAs to non‐GLP1RA anti‐obesity medications (AOMs), (5) weight change and variables associated with weight change during GLP1RA therapy, and (6) frequency and causes of emergency department (ED) visits and hospitalizations during the study period.

Gaps between prescriptions were permitted if a new prescription was filled within 30 days of the weight measurement. Attribution of ED visits and hospitalizations to medication use was determined based on diagnosis codes associated with each encounter and their consistency with known adverse effects of GLP1RAs. A full list of diagnosis codes used to classify these events is provided in Appendix S1.

### 2.9 Covariates and planned analyses

Key covariates included age, sex, race/ethnicity, baseline weight, body mass index (BMI) and presence of comorbidities such as type 2 diabetes, hypertension, or dyslipidaemia. These will be adjusted for in multivariable models evaluating weight change and persistence outcomes. Planned subgroup analyses include comparisons between semaglutide and tirzepatide users, stratified analyses by sex and BMI class, and sensitivity analyses including patients with treatment gaps >84 days.

### 2.10 Ethics approval

This study was approved by the Vanderbilt University Medical Center Institutional Review Board (IRB# 250171). A waiver of informed consent was granted due to the retrospective nature of the study and minimal risk involved. All data were de‐identified in compliance with HIPAA regulations.

### 2.11 Statistical analysis

Patient demographics, comorbidities, medication information and weight data were summarized using descriptive statistics. Percent total weight loss was compared between'low‐dos' and' high‐dos' groups at 3, 6, 9 and 12 months of persistent medication use using Wilcoxon Rank Sum tests. Linear mixed‐effects models were used to examine the trajectory of weight change over time while on medication. Fixed effects included age, sex, baseline weight, medication at 9 months, dose group at 9 months, and time since medication initiation, treated as a restricted cubic spline with three knots to account for potential non‐linearities in the effect of time. Interactions between dose group and medication, as well as between dose group and time since medication initiation, were included. A random intercept accounted for individual differences in baseline weight, and a random slope for time since medication initiation allowed the rate of weight change to vary across patients. Partial effects plots were used to visualize the relationships between key predictors and weight, and contrasts were applied to assess specific group differences. The analysis was restricted to patients with either a'low‐dos' or'high‐dos' at 9 months. Complete‐case analysis was performed. All statistical analyses were conducted using R version 4.2.2.

## 3 RESULTS

### 3.1 Baseline characteristics

During the study period, 2855 patients were identified as having enrolled in the bundle, of whom 2306 (81%) received at least one prescription for semaglutide or tirzepatide. The median age of the cohort was 46 years (IQR 38–55 years), and 85% were female (Table 1). The cohort was predominantly White (68%), with 28% identifying as Black and 4% as other. Only 4% identified as Hispanic. The most common baseline comorbidities were hypertension (43%), followed by osteoarthritis (32%) and gastroesophageal reflux (28%). Thirteen percent had pre‐existing type 2 diabetes.

**TABLE 1:** Cohort demographics and baseline comorbidities.

	Overall
	(*N* = 2306)
Age at medication initiation	
Mean (SD)	45.9 (11.1)
Median [Q1, Q3]	46.0 (38.0, 55.0)
Sex	
Female	1965 (85%)
Male	341 (15%)
Race	
Black	629 (27%)
White	1569 (68%)
Other	88 (4%)
Missing	20 (1%)
Ethnicity	
Hispanic	100 (4%)
Not Hispanic	2022 (88%)
Missing	184 (8%)
Comorbidities	
Smoking	92 (4%)
VTE	55 (2%)
GERD	655 (29%)
NAFLD	164 (7%)
Hyperlipidemia	622 (27%)
CKD ≥III	55 (2%)
Solid organ transplant	7 (0.3%)
Congestive heart failure	39 (2%)
Myocardial infarction	64 (3%)
Stroke	20 (1%)
Obstructive sleep apnea	549 (24%)
Osteoarthritis	746 (32%)
Type 2 diabetes	296 (13%)
Hypertension	1001 (43%)
Asthma or COPD	376 (16%)
Any comorbidity	1860 (81%)

Abbreviations: CKD ≥III, chronic kidney disease Stage 3 or greater; COPD, chronic obstructive pulmonary disease; GERD, gastroesophageal reflux disease; NAFLD, non‐alcoholic fatty liver disease; Q1, first quartile; Q3, third quartile; VTE, venous thromboembolism.

### 3.2 Medication prescribing

The vast majority of patients (92%) initiated semaglutide as the first medication after enrolling in the bundle (Table 2). Consequently, 70% of patients received semaglutide exclusively, 5% received tirzepatide only and 25% received both medications at different times. Patients received a median of 12 prescriptions (IQR 6–17) and a median of 3 (IQR 2–5) unique prescriptions (i.e., either new dose or drug change). Using a 3‐supply gap (84 days) to define discontinuation, the median duration of use within the cohort was 10.7 months (IQR 5.4–16.3). Medication discontinuation was 14%, 24%, 35% and 50% at 3, 6, 9 and 12 months, respectively. Only 9.1% of patients were using a non‐GLP1RA AOM prior to starting semaglutide or tirzepatide, and only 5.7% newly initiated a non‐GLP1RA AOM after discontinuing either semaglutide or tirzepatide.

**TABLE 2:** Prescribing of semaglutide and tirzepatide.

	Overall (*N* = 2306)
Medication persistence (median (Q1, Q3) months)	10.7 [5.4, 16.3]
Number of GLP1 prescriptions	12.0 [6.0, 17.0]
Number of unique GLP1 prescriptions* (Median [Q1, Q3])	3.0 [2.0, 5.0]
GLP1 taken at initiation	
Semaglutide	2126 (92.2%)
Tirzepatide	180 (7.8%)
GLP1s taken during study period	
Semaglutide only	1614 (70.0%)
Tirzepatide only	117 (5.1%)
Both drugs	575 (24.9%)
Semaglutide	Overall (*N* = 2189)
0.25 mg	418 (19.1%)
0.25/0.5 mg	1631 (74.5%)
0.5 mg	324 (14.8%)
1 mg	1651 (75.4%)
1.7 mg	661 (30.2%)
2 mg	986 (45.0%)
2.4 mg	501 (22.9%)
Any dose ≥1 mg	1783 (81.5%)
Any dose ≥2 mg (any high dose)	1206 (55.1%)
Tirzepatide	Overall (*N* = 692)
2.5 mg	210 (30.3%)
5 mg	237 (34.2%)
7.5 mg	271 (39.2%)
10 mg	367 (53.0%)
12.5 mg	360 (52.0%)
15 mg	196 (28.3%)
Any dose ≥10 mg (any high dose)	519 (75.0%)

Abbreviations: GLP1, glucagon like peptide 1 receptor agonist; mg, milligrams; Q1, first quartile; Q3, third quartile.

### 3.3 Semaglutide and tirzepatide dose titration

**FIGURE 1:**
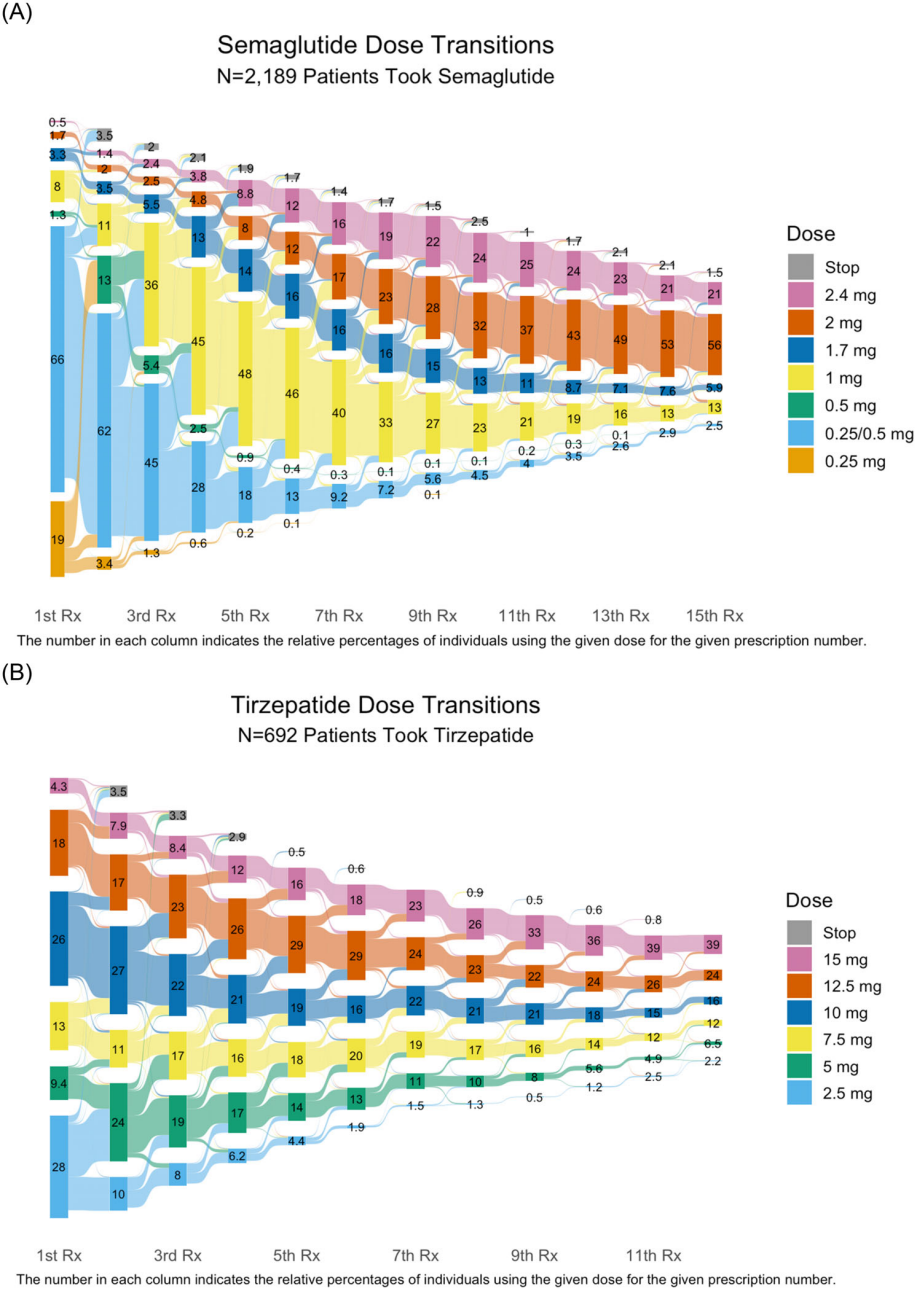
Sankey diagram of (A) semaglutide prescribing and (B) tirzepatide prescribing over the first 15 prescriptions.

Transitions between doses of semaglutide and tirzepatide are illustrated in the Sankey diagram in Figure 1A,B, respectively. Of the 2189 patients who received semaglutide, 93% of patients initiated on 0.25 mg or 0.25/0.5 mg starter pack. By the 6th prescription, 24% were receiving 2 mg or more, and 12% received 2.4 mg of semaglutide. For the 692 who received tirzepatide, likely owing to switching between GLP1RAs, 48% of patients initiated at 10 mg or higher, while 28% initiated on 2.5 mg. By the 6th prescription, 63% of participants received 10 mg or higher, and 18% received 15 mg of tirzepatide; 81% of patients received one prescription of ≥1 mg and 55% received ≥2 mg of semaglutide; 75% of patients received at least one prescription of ≥10 mg of tirzepatide, while 28% received 15 mg.

**FIGURE 2:**
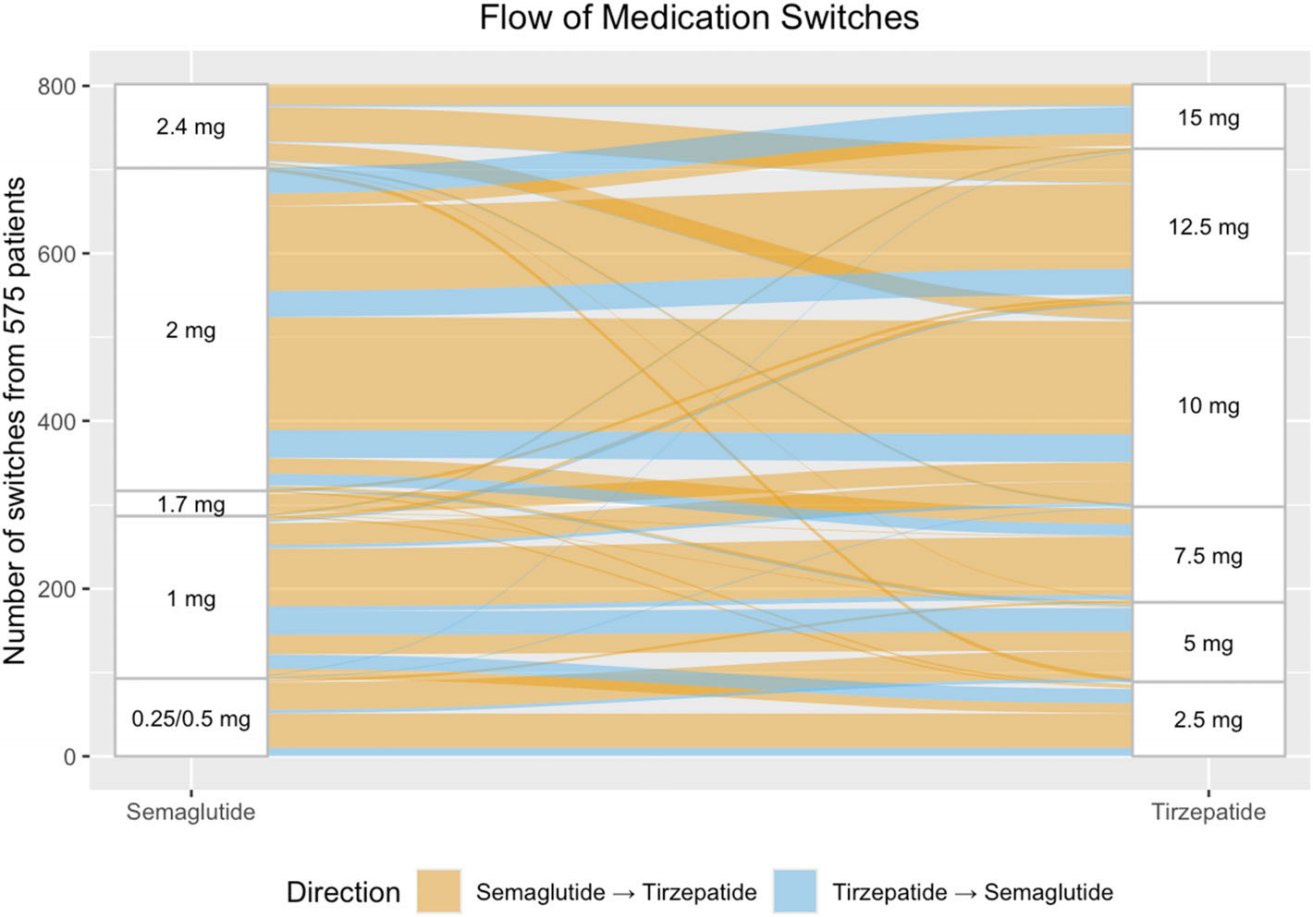
Flow of switches between semaglutide and tirzepatide.

Medication switches were common among the group, with 575 (25%) using both semaglutide and tirzepatide at different points of the study period. In total, 18% had 1 medication switch, and 5% switched twice (Figure 2). The most common transitions were semaglutide 2 mg to tirzepatide 10 mg (135 patients, 23%), followed by semaglutide 2 mg to tirzepatide 12.5 mg (101 patients, 18%), and semaglutide 1 mg to tirzepatide 7.5 mg (69 patients, 12%).

### 3.4 Emergency room visits and hospitalizsations

At least one emergency room visit and hospitalization related to medication occurred in 13.6% of the overall cohort. The most common reason for ER visits and hospitalizations in this cohort was for GI disorders (8.6% of visits) which include gastroesophageal reflux, constipation and gastroenteritis, followed by abdominal pain (2.4% of visits), and nausea, dehydration and nutritional issues (2.3%). Hepatobiliary‐related complications were rare (0.2% of visits), and one visit for acute pancreatitis was identified.

### 3.5 Weight changes

For patients with at least 3 months of persistent GLP1RA use, %TWL was 5.5% (IQR 3.0–8.0%). For those with 6 months persistent use, %TWL was 9.4% (IQR 6.0–13.4%); for 9 months, %TWL was 12.9% (IQR 8.4–17.1); and for 12 months persistent use, %TWL was 14.4% (9.5–20.5%). When comparing those who achieved a ‘low‐dose’, or ‘high‐dose’ of semaglutide or tirzepatide at specific time points, on univariate analysis we found no difference in %TWL between those receiving low‐ or high‐dose semaglutide or tirzepatide at 6, 9 and 12 months of persistent use (*p* = 0.3, 0.6, and 0.8, respectively).

We also evaluated patients who used only a single GLP1RA (i.e., did not switch GLP1RAs). For patients who received semaglutide exclusively (*N* = 1614), %TWL was 5.7% [IQR 3.2–8.0], 9.8% [IQR 6.4–13.6], 13.5% [IQR 9.0–18.4], and 15.6% [10.8–21.9] at 3, 6, 9 and 12 months respectively. Follow‐up rate at each time‐point for semaglutide users was 62%, 47%, 37% and 29%, respectively. For those who received tirzepatide exclusively (*N* = 117), %TWL was 7.1% [4.4–9.7], 11.9% [7.6–16.6], 16.3% [12.0–22.3], and 14.1 % [9.9–22.1] at 3, 6, 9 and 12 months, respectively. Follow‐up rate at each time‐point for tirzepatide users was 63%, 56%, 40% and 14% respectively.

Weight loss appeared to be maintained with a percent change in weight of 0.3% and 0.1% at 3 and 6 months post‐GLP1RA, respectively, although missingness was high for weight data after discontinuation (>65% for both timepoints). For patients remaining on medication at given timepoints, missingness of weight data was 19%, 22%, 20% and 14% at 3, 6, 9 and 12 months, respectively, and 36.8% lacked weight data within 30 days of medication discontinuation.

### 3.6 Factors associated with weight loss

**FIGURE 3:**
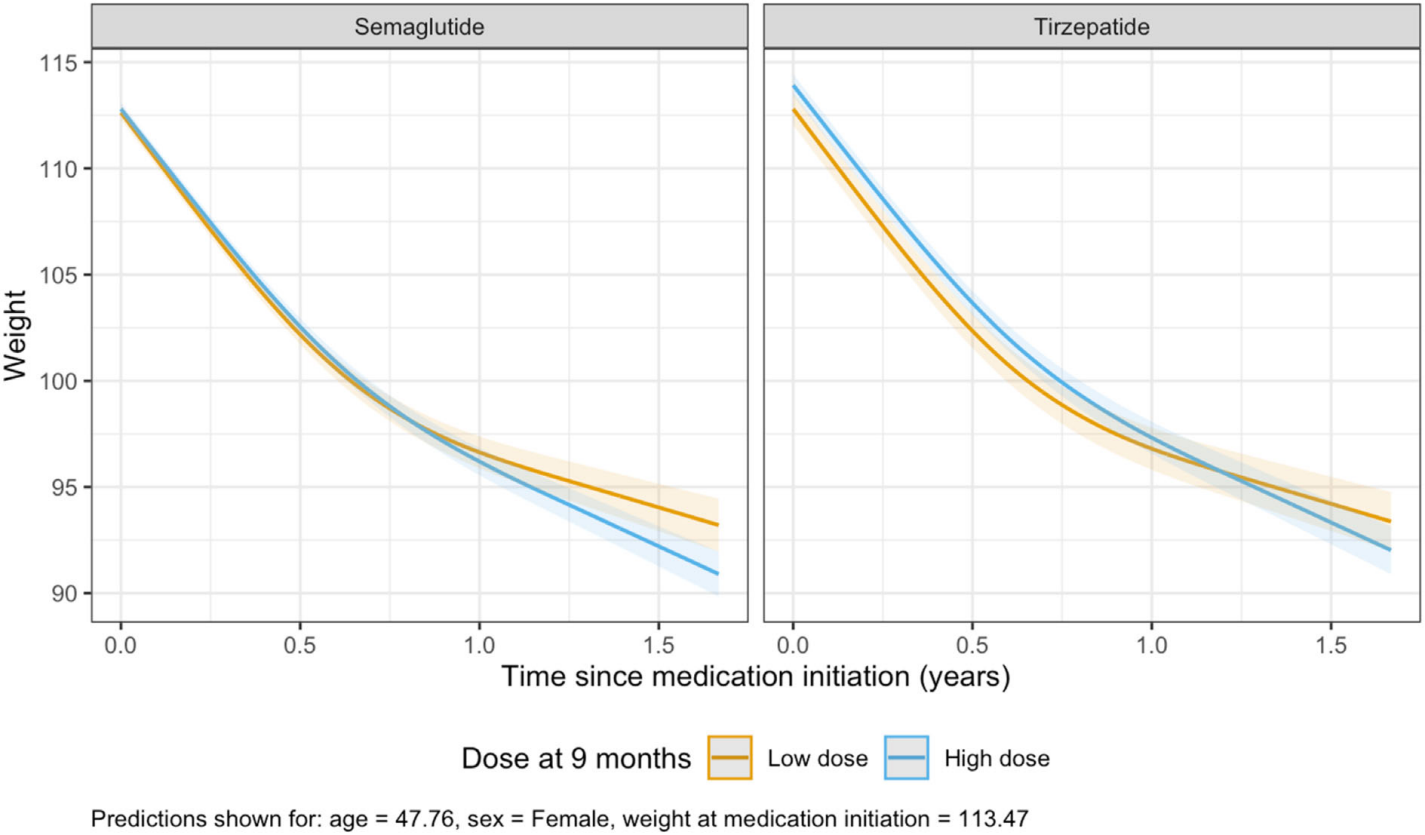
Change in weight over time by ‘low‐dos’ versus ‘high‐dos’ at 9 months.

In multivariable linear mixed‐effects models examining weight over time among patients with dose information at 9 monthspost‐drug initiation, weight decreased significantly over time (*p* < 0.001). Male sex was associated with an estimated 0.89 kg lower weight compared with females (95% CI −1.46 to −0.33, *p* = 0.002), and higher baseline weight was associated with higher weight across follow‐up (estimate: 0.98 kg; 95% CI 0.97–0.99, *p* < 0.001). The interaction between high‐dose treatment and time since medication initiation was significant (*p* < 0.001), indicating that patients on high‐dose regimens by 9 months experienced greater initial weight loss, though the rate of loss slowed over time (Figure 3). Model‐estimated contrasts indicated that high‐dose users at 9 months experienced greater total weight loss compared with low‐dose users, with estimated losses of 14.7 kg (95% CI 14.1–15.3) versus 13.6 kg (95% CI 12.9–14.3) per year, respectively. This difference of 1.1 kg was statistically significant (*p* = 0.020). The interaction between high‐dose treatment and time since medication initiation was significant (*p* < 0.001), indicating that patients on high‐dose regimens by 9 months experienced greater initial weight loss, though the rate of loss slowed over time (Figure 3). Model‐estimated contrasts indicated that high‐dose users at 9 months experienced greater total weight loss compared with low‐dose users, with estimated losses of 14.7 kg (95% CI 14.1–15.3) versus 13.6 kg (95% CI 12.9–14.3) per year, respectively. This difference of 1.1 kg was statistically significant (*p* = 0.020).

## 4 DISCUSSION

While the landmark STEP and SURMOUNT trials established the efficacy of semaglutide and tirzepatide for weight loss,^1^, ^2^ translating this efficacy into real‐world effectiveness has been hampered by challenges including medication tolerability, adherence and continued access. This study examined prescribing patterns, persistence and weight outcomes among patients initiating these GLP1RAs within a specialized medical weight management programme with consistent insurance coverage of semaglutide and tirzepatide. We observed longer medication persistence compared with previous real‐world reports, although difficulties in achieving optimal dose titration persisted. Notably, the weight loss observed in our cohort approached levels reported in clinical trials, potentially reflecting the impact of management within a dedicated obesity medicine setting with structured support and prolonged persistent usage.

The clinical trials leading to semaglutide and tirzepatide approval reported a remarkably low discontinuation rate of 7%–11%, but these rates of low discontinuation have not been replicated in the real‐world setting.^1^, ^2^ Prior real‐world studies have consistently highlighted low persistence rates with GLP1RAs used for weight management.^16^ Weiss et al. reported 65% adherence at 12 months among patients with type 2 diabetes receiving various GLP1RAs,^9^ while Gleason et al. found over half of patients without diabetes discontinued semaglutide by 12 months.^17^ Studies focusing on tirzepatide by Hankosky et al. reported median times to discontinuation as short as 60 days, with 6‐month persistence rates of 68–74%.^18^, ^19^ Similarly, our cohort demonstrated a median persistence duration of 10.7 months (IQR 5.4–16.3), with 50% of patients discontinuing therapy by 12 months. The high discontinuation rate by 12 months may reflect continued supply challenges, toleration difficulties or a desire to discontinue medications once weight loss has been achieved.

One potential explanation for higher persistence in our study is more advanced patient support systems. As an obesity specialty clinic, all patients receive multidisciplinary care including visits with dietitians and, if needed, licensed psychologists and social workers. Patients who enrol in the system‐wide patient portal can receive pharmacy refill reminders and have access to system‐run pharmacies that integrate with the patient portal, allowing for ease of messaging to providers requesting refills or to submit concerns regarding drug tolerance. These differences may limit generalizability to less resource‐intensive primary care settings.

In addition, challenges with dose titration, crucial for maximizing efficacy as shown in the SURMOUNT and SURPASS trials,^2^, ^20^ were evident in our cohort, mirroring findings from other real‐world settings. Ladebo et al. found only 25% of Danish patients using semaglutide reached the 2.4 mg dose.^12^ Similarly, we found only 22% of our patients received at least one prescription for 2.4 mg semaglutide, although 55% reached 2 mg or higher. For tirzepatide, previous studies reported that only 23%–44% of patients received doses of 10 mg or higher, and fewer than 10% reached 15 mg within approximately 6 months.^18^, ^19^, ^21^ Our findings were more favourable, with 75% achieving at least 10 mg and 28% receiving at least one 15 mg prescription, yet still indicate that a substantial portion of patients do not reach maximal dosing even under specialized care. Medication switches between semaglutide and tirzepatide were also common (29% of users), often occurring from higher doses of semaglutide to intermediate or higher doses of tirzepatide, suggesting attempts to manage tolerability, optimize response, or maintain treatment during supply shortages. Unfortunately, as reasons for medication switches were not documented as part of routine care, nor were they documented in an objective, categorical manner, this study is unable to specify and investigate the reasons for medication switching.

Regarding effectiveness, previous real‐world studies, often limited by size or follow‐up duration, reported 6‐month weight loss ranging from 11% to 13% for semaglutide or tirzepatide.^18^, ^19^, ^22^ Our cohort achieved a median weight loss of 9.4% at 6 months and, importantly, 14.4% at 12 months among persistent users, approaching the results observed in the STEP and SURMOUNT trials. Intriguingly, the impact of dose on degree of weight loss seems minimal. While greater weight loss was noted at 9 months, the difference was clinically insignificant. This finding should be interpreted cautiously, particularly given data missingness at later time points, and may reflect confounding factors (e.g., patients achieving sufficient response at lower doses) rather than a true lack of dose–response in practice. Data on weight after medication discontinuation suggested minimal regain at 3 and 6 months, but these findings are limited by very high rates of missing data (>65%). The high rate of missingness may reflect patients discontinuing weight loss treatment and follow‐up visits due to perceived lack of effectiveness, achievement of personal weight loss goals, or external factors such as relocation or insurance changes related to employment transitions. Furthermore, emergency room visits and hospitalizations potentially related to these medications were infrequent, with gastrointestinal issues being the most common reason, consistent with the known side‐effect profile.

Despite this study taking place in a resource‐rich, specialized multidisciplinary obesity clinic, a significant proportion of patients did not follow the recommended titration defined in the package inserts for semaglutide and tirzepatide.^5^, ^15^ Due to the retrospective nature of this study, reasons for patients not following the recommended titration could not be directly determined. Although patients were cared for by obesity medicine specialists with knowledge of the required titration and supported by pharmacists who assist with ensuring appropriate titration, this poor adherence could reflect systems‐level issues that fail to ensure titration adherence due to the frequency of required dose adjustments. Other factors could explain this finding, including medication supply issues, intolerance of higher doses, or patients desiring to continue at a dose perceived to be effective. Future studies should explore reasons for deviations from the recommended titrations.

This study has several limitations inherent to its observational design. First, several factors exist in our study that may limit generalizability to the broader primary care settings. The findings originate from a single, specialized academic weight management centre, potentially enrolling patients with higher motivation or specific clinical characteristics, and patients were enrolled in a ‘no cost to the patient’ value care program which may impact medication usage patterns and discontinuation compared with insurance coverage programs that require a co‐pay for medications. Additionally, significantly more participants used semaglutide instead of Tirzepatide, as much of the study period precluded Tirzepatide's approval for obesity treatment. Reflecting the demographics of our clinic, our study primarily enrolled White, female patients, limiting generalizability to other populations. Although prescription fill data from the clearinghouse provides objective evidence of medication dispensing, it does not confirm medication administration. Early discontinuations or difficulties with titration could stem from various factors not directly captured, including medication intolerance, drug supply shortages, or patients achieving their desired weight goals and electing to stop treatment as a result. The patients discontinuing therapy and not providing weight data, particularly at 12 months and after discontinuation, limits the robustness of the weight loss analyses and conclusions about weight maintenance. Despite these limitations, this study provides valuable real‐world data on a large cohort using contemporary GLP1RAs within a supported environment, linking prescription data with EHR information.

To address these limitations, future studies should employ prospective studies of real‐world users of GLP1RAs utilizing a combination of EHR‐based data collection which is enriched with non‐routine data collection such as at‐home weight collections using internet‐connected scales and remote survey collection documenting medication usage, real‐world adverse event rates and discontinuation rates and reasons. Future studies should also explore how real‐world usage differs in particular subsets of patients utilizing GLP1RAs such as patients with mental illness or of differing socioeconomic statuses.^23^, ^24^ We also note that future studies should expand on the cost implications of GLP1RAs given their heightened cost balanced with the potential for reduction of costs treating obesity‐related conditions.^25^, ^26^

## 5 CONCLUSION

In a cohort of patients managed within a specialized weight loss programme with facilitated insurance access, persistence with semaglutide and tirzepatide therapy significantly exceeded rates reported in previous real‐world studies. However, achieving recommended target doses remains a challenge for many patients. The observed 9 and 12‐month weight loss approached levels seen in clinical trials, suggesting that structured clinical support and consistent medication access can enhance the real‐world effectiveness of these agents. Nonetheless, difficulties in optimal dose titration persist even in specialized settings and warrant further investigation. Future research should explore strategies to overcome titration barriers and confirm these findings in broader patient populations and healthcare settings, especially as medication supply issues resolve and newer agents become available.

## FUNDING INFORMATION

This publication was supported by CTSA award No. UL1 TR002243 from the National Center for Advancing Translational Sciences. Its contents are solely the responsibility of the authors and do not necessarily represent official views of the National Center for Advancing Translational Sciences or the National Institutes of Health.

## CONFLICT OF INTEREST STATEMENT

The authors report no financial conflicts of interest.

## PEER REVIEW

The peer review history for this article is available at https://www.webofscience.com/api/gateway/wos/peer‐review/10.1111/dom.70004.

## Supporting information


**Figure S1:** Flow diagram.

**Appendix S1.** Supporting Information.

## Data Availability

The data that support the findings of this study are available on request from the corresponding author. The data are not publicly available due to privacy or ethical restrictions.
