# Supplementary figures and images for: Real‐world titration, persistence & weight loss of semaglutide and tirzepatide in an academic obesity clinic
Source: Diabetes Obes Metab. 2025 Aug 5;27(11):6200–9. doi: 10.1111/dom.70004 (PMC12515774; doi:10.1111/dom.70004)

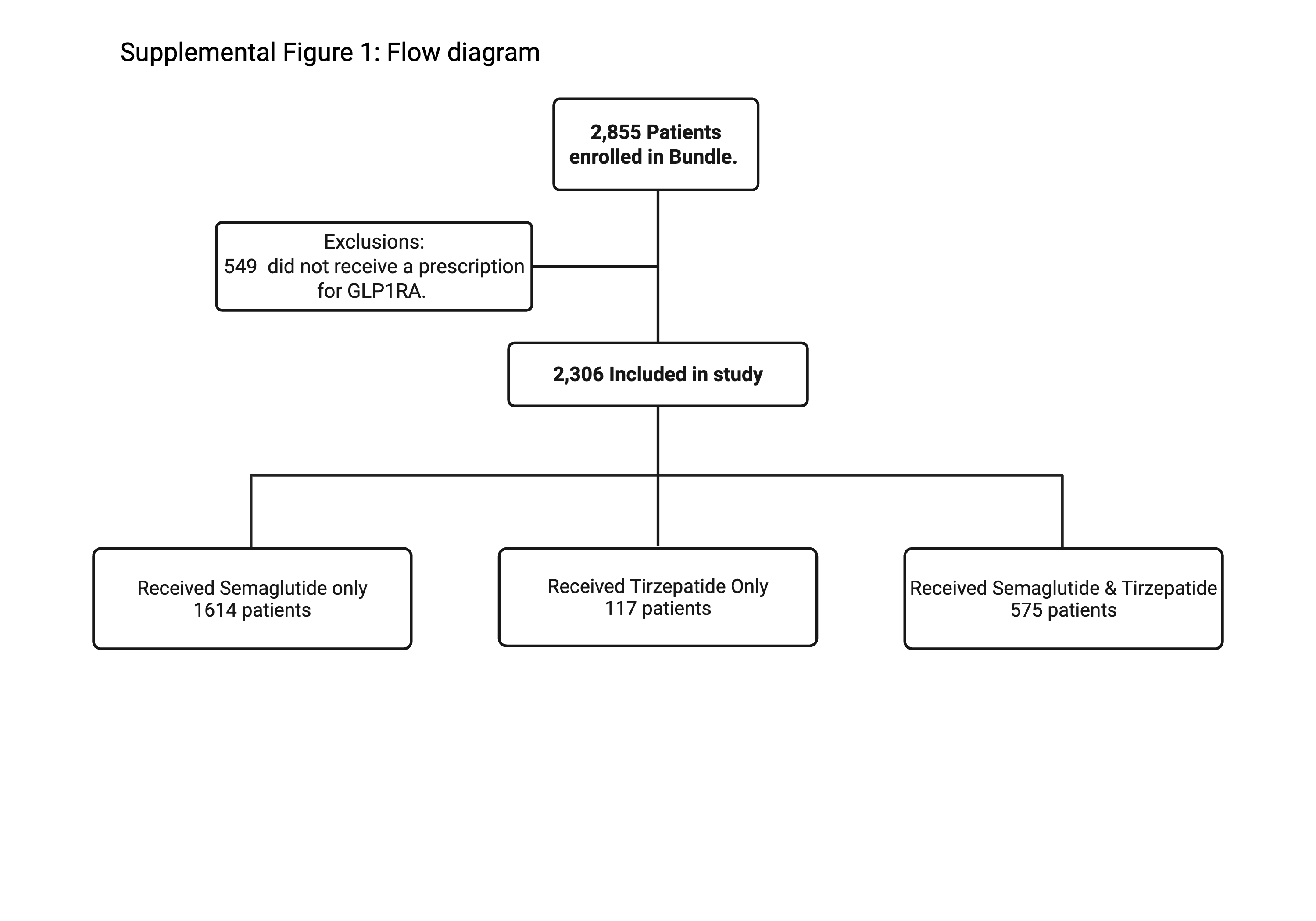

Supplement: Supplementary file 1 — Figure S1: Flow diagram. [file DOM-27-6200-s002.jpeg]
